# Intravitreal Transplantation of Human Umbilical Cord Blood Stem Cells Protects Rats from Traumatic Optic Neuropathy

**DOI:** 10.1371/journal.pone.0069938

**Published:** 2013-08-05

**Authors:** Bing Jiang, Pu Zhang, Dan Zhou, Jun Zhang, Xiang Xu, Luosheng Tang

**Affiliations:** 1 Department of Ophthalmology, Second Xiangya Hospital, Central South University, Changsha, China; 2 Shenzhen Beike Cell Engineering Research Institute, Shenzhen, China; Dalhousie University, Canada

## Abstract

**Objectives:**

To treat traumatic optic neuropathy (TON) with transplantation of human umbilical cord blood stem cells (hUCBSC) and explore how transplanted stem cells participate in the neuron repairing process.

**Methods:**

A total of 195 Sprague-Dawley rats were randomly assigned to three groups: sham-surgery, optic nerve injury, and stem cell transplant group. Optic nerve injury was established in rats by directly clamping the optic nerve for 30 seconds. hUCBSC was microinjected into the vitreous cavity of injured rats. Optic nerve function was evaluated by flash visual evoked potentials (F-VEP). Apoptosis in retina tissues was detected by TUNEL staining. GRP78 and CHOP gene expression was measured by RT-PCR.

**Results:**

After injury, transplantation of hUCBSC significantly blunted a reduction in optic nerve function indicated by smaller decreases in amplitude and smaller increases in peak latency of F-VEP waveform compared to the injury alone group. Also, significant more in retinal ganglion cell (RGC) count and less in RGC apoptosis were detected after transplantation compared to injured rats. The protective effect correlated with upregulated GRP78 and downregulated CHOP mRNA expression.

**Conclusion:**

Intravitreal transplantation of hUCBSCs significantly blunted a reduction in optic nerve function through increasing RGC survival and decreasing retinal cell apoptosis. The protective role of transplantation was associated with upregulation of GRP78 expression and downregulation of CHOP expression in retinal cells.

## Introduction

Traumatic optic neuropathy (TON) is an important cause of severe vision loss in 0.5 to 5% of patients with closed head trauma [1]. Usually, trauma causes immediate mechanical damage to a fraction of retinal ganglion cell (RGC) axons, which becomes irreversible during subsequent RGC degeneration. Following the initial damage to the optic nerve, swelling within the optic nerve canal or compression by bone fragments may lead to secondary RGC loss [2]. This secondary damage further impairs the already compromised blood supply to surviving RGCs, and subsequently causes apoptotic cell death [2]. RGCs are specialized cells within the optic nerve and form a key part of an intricate chain responsible for transmitting information from the eye to the vision centers within the brain. It is therefore a promising strategy to limit these secondary mechanisms and preserve surviving RGCs to reduce vision loss. A variety of treatments including conservative management, steroids application, surgical decompression, and a combination of surgery and steroid treatment have been used to prevent pathological damages to the optic nerves and increase retinal ganglion cell (RGC) survival post trauma. However, no treatment has been proven to be particularly effective in the treatment of TON [3]. In contrast, using stem cells to replace lost neuron cells is a promising strategy that has been developed recently.

The hypothesis that neurogenesis contributes to functional recovery of brain injury stimulated attempts to transplant stem cells systemically or locally to replace lost neurons at the site of injury. Human umbilical cord blood stem cells (hUCBSCs) have become a potential for treating conditions ranging from ischemic injury to neurodegenerative diseases due to their advantages in terms of clinical transplantation. These advantages include that the cord blood can be noninvasively harvested at birth, its high quality unaffected by aging or postnatal viral infection, as well as the lack of ethical issues currently surrounding the use of hUCBSCs [4]. The role of hUCBSC transplantation in neurodegenerative diseases has been widely investigated in animal models. For instance, intravenous or intraperitoneal administration of hUCBSC reduced the severity of neurological deficits caused by middle cerebral artery occlusion [5]. Also, intravenous administration of hUCBSC improved the functional state of the brain and minimized behavioral deficits in rats suffering from hemorrhagic or traumatic brain and spinal cord injury [6]. Infusion of hUCBSC delayed the progression of amyotrophic lateral sclerosis and increased the lifespan of diseased transgenic mice [7]. Even with so much promising data, it is still unclear how transplanted stem cells participate in the neuron repair process. Most studies believe that cord blood cells can turn into brain cells i.e. neurons, astrocytes, oligodendrocytes, endothelial cells and microglia to replace the lost cells [8]. However, even now this mechanistic view does not match experimental findings. In contrast, several studies reported that only a few transplanted human umbilical cord cells were detected in the injured tissue of grafted animals [9]. Particularly, rapid improvement in brain function within a few days of human cord blood cell being transplanted into animals may indicate that mechanisms other than cell replacement are of primary importance in these cases [4]. Possible mechanisms include that transplanted hUCBSCs may repair brain damage via releasing neurotrophic factors as well as producing a variety of cytokines and chemokines, creating a favorable microenvironment for increased cell survival [10].

Recently, the endoplasmic reticulum (ER) stress was found to play a role in the death of RGCs. Endoplasmic reticulum is where proteins are processed, which includes glycosylation, folding, oligomerization, and disulfide processes. After a series of physiological and biochemical stimulation, unfolded protein accumulates in the ER lumen, which is associated with various neurodegenerative diseases such as Alzheimer's, Huntington's, and Parkinson's diseases [11]. However, the role of ER stress in RGC injury has not yet been fully elucidated. The molecular chaperone protein GRP78 (glucose-regulated protein 78) and CHOP (C/EBP homologous protein) are classic ER stress markers. GRP78, also known as immunoglobulin heavy chain binding protein and heat shock protein 70 homologous, is an important endoplasmic chaperone. GRP78 plays a protective role in ER stress through binding and transporting the unfolded protein [12]. CHOP is a proapoptotic gene and its basic expression is lower under normal circumstances [13]. However, whether the transplanted hUCBSCs can reduce ER stress to participate in the neuron repair process remains unreported.

In this study, we transplanted hUCBSCs by microinjection of cells into rat vitreous cavity to protect the optic nerve from injury caused by direct clamping of the optic nerve. We investigated the protective mechanism by examining neuron apoptosis, GRP78, and CHOP expression in hUCBSC transplanted retina compared to the injured retina without transplantation.

## Materials and Methods

### Preparation of Human umbilical cord blood stem cells

The study protocol was approved by the university ethics committee (The Second Xiangya Hospital of Central South University Review Board). Donors were fully informed about the use of their umbilical cord blood in the study. Written informed consent forms were obtained from all donors. The human umbilical cord blood stem cells (hUCBSCs) were prepared from umbilical cord blood of 5 healthy donors as previously described [4]. Briefly, human umbilical cord blood was collected in accordance with the sterile procurement guidelines. After collection, human umbilical cord blood was diluted with saline at 2∶1 and 30 ml of the diluted UCB was then loaded on the surface of 15 ml Ficoll in each 50 ml centrifuge tube and then centrifuged (750 g×22 minutes). Mononuclear cells were collected and washed twice with saline. Contaminated erythrocytes were lyzed with lysis buffer. A 2∼6×10^6^/ml of purified mononuclear cells was seeded in 10 cm flask containing 10 ml of DMEM/F12 culture medium supplemented with 20 ng/ml of bFGF and EGF, and 2% (v/v) B-27 Stem Cell Culture Supplements. Cells were cultured at 37°C, 5% CO_2_. Cell growth was regularly monitored and non-qualifying cells were eliminated. The highly homogeneous cells possess a round shape and non-adherence to the flask. Cells were harvested for experiment when cell quantity was ≥1×10^7^, viability was ≥95%, and contained 1.0–2.0% CD34^+^ cells. For quality control, cells were tested for mycoplasma, endotoxin, infection of HBV, HCV, HIV, ALT, and syphilis. No positive sign was found. For decreasing the divergences between donors, a mixture of cells was applied to the animal experiments.

### Animals

A total of 195 Sprague-Dawley rats, weighting 220–270 g, were provided by Experimental Animal Center, Central South University. Animals were housed in a controlled environment (24°C, 60% humidity) with food and water *ad libitum*. All experiments were conducted in accordance with the National Institutes of Health Guide for the Care and Use of Laboratory Animals and the Chinese legislation on the use and care of laboratory animals. The animal protocol was approved by the Animal Use Committee of Central South University. All efforts were made to minimize animal suffering, to reduce the number of animals used, and to utilize alternatives to in vivo techniques.

### Experimental models

Animals were randomly assigned to three groups: sham-surgery group (n = 25), TON model (optic nerve injury) group (n = 85), and hUCBSC transplantation group (n = 85). In the TON model and transplantation groups, traumatic optic neuropathy was established by clamping the optic nerve directly. Briefly, rats were intraperitoneally anesthetized (10% chloral hydrate, 3.5 ml/kg) and the upper eyelid skin and bulbar conjunctiva were vertically cut under a microscope to expose the optic nerve. The injury was conducted by directly clamping the optic nerve with 40 g of constant holding force for 30 seconds at 2 mm behind the eyeball. The incision was then sutured. In the sham-surgery group, surgery was performed but without optic nerve injury. In the transplantation group, 10-μl (10^6^) hUCBSCs were microinjected into rat vitreous cavity using 30 g syringe needles under the microscope immediately 30 min after optic nerve injury. As injection control, animals in sham-surgery and TON model group were injected with saline.

### F-VEP measurement

The flash visual evoked potentials (F-VEP) were recorded according to international standards of clinical visual electrophysiology using the visual electrophysiology system (Chongqing Medical Equipment Factory, China). Briefly, rats were anesthetized and fixed to the experiment station. Pupils were dilated with amide tropicamide. The recording electrode was subcutaneously inserted into the skin at the occipital protuberance, while the reference electrode was inserted into the skin at the midpoint of the eyes, and the ground electrode was inserted into the skin behind the right ear. After rats were allowed to adapt to the dark for 15 min, F-VEP was recorded 1 h, 7 d, 14 d, 21 d and 28 d after surgery. Three stable waveforms were recorded for each animal for both the normal and injured eyes. The recorded F-VEP waveform is a relatively stable N-P-N wave, named N1, P1, N2. The latency of initial N1, P1 and N2 waves are recorded as milliseconds (ms) while amplitude of N1-P1 and P1-N2 was recorded as the micro-volts (μV). The peak latencies of N1 were measured from the beginning to the peak of N1. The peak latencies of P1 were measured from the beginning to the bottom of the P1 peak, and so on. The amplitude value of N1-P1 was from N1 peak to the bottom of P1, and so on. Each index was measured 3 times and averaged.

### Tissue preparation

Five rats were sacrificed at 3 d, 7 d, 14 d, 21 d and 28 d post-surgery and retinas were removed, fixed in paraformaldehyde, and used for hematoxylin-eosin staining. Five rats were sacrificed at 3 h, 12 h, 24 h, 48 h, 72 h and 7 d post-surgery and fresh retinas were used for frozen sections and TUNEL detection of apoptotic cells. Five rats were sacrificed at 3, 12, 24, 48, 72 hrs and 7 days post injury for total RNA isolation and RT-PCR analysis of GRP78 and CHOP mRNA expression.

### Hematoxylin-eosin staining

Sections were dipped into gradient ethanol, followed by hematoxylin staining for 10 minutes, rinsed in 1% (v/v) hydrochloric acid/alcohol for 5–10 seconds, incubated in saturated lithium carbonate solution for 5–10 seconds, and stained with eosin for 10 minutes. The slides were rinsed with dH_2_O between each step. The slides were then dehydrated with gradient ethanol, cleared with xylene, mounted with neutral gum, dried, and observed under light microscopy. RGC was counted by two blinded investigators who were unaware of the experiment design. Briefly, under a high magnification (40× objective), RGCs were counted from 3 fixed views which are 50, 100, and 150 micron from the optic nerve center and the RGC amounts from 3 views were averaged.

### TUNEL staining

The eye-embedded sections were digested with proteinase K for 10 minutes and then rinsed with 3% H_2_O_2_ in methanol for 10 min to block endogenous peroxidase. After the sections were washed with PBS, they are permeabilized with 0.1% Triton X-100 in 0.1% sodium citrate for 2 min on ice, then incubated with terminal deoxynucleotidyl transferase-mediated nick end labeling reaction mixture for 60 min at 37°C. After the slides were washed with PBS, converter-POD was added and the section was incubated for 30 min at 37°C in a humidified chamber. Around 50–100 µl of 3,3′-diaminobenzidine-substrate solution was dropped onto the slides and analyzed under a light microscope after incubating for 10 min. Percentage of positive cells (nuclei with brown particles) was calculated [positive cell ratio  =  (positive cells/total cells counted) ×100% ].

### RT-PCR

After sacrifice, the eyes were removed and retina was separated. Total RNA was isolated by using Trizol reagents (Invitrogen, Carlsbad, CA, USA) and reverse-transcribed by using RevertAid™ First Strand cDNA Synthesis Kits (Fermentas, Hanover, MD, USA). The mRNA expressions of GRP78, CHOP, and β-actin were amplified and the band-pixel value was analyzed by Gel doc2000 gel image analysis system (Bio-Rad, Hercules, CA, USA). The GRP78 and CHOP pixel intensity quantifications were normalized at each time point to beta-actin pixel intensity.

### Statistical analysis

Data was presented as mean and standard error of the mean, and analyzed using the statistical package for the Social Science Version 19.0. Data was analyzed using a multivariate 2-way ANOVA with post-hoc tests that control for multiple comparisons. A *p*<0.05 was considered statistically significant.

## Results

### hUCBSC transplantation improved the flash visual evoked potentials (F-VEP) waveform of optic nerve injured rats

**Figure 1:**
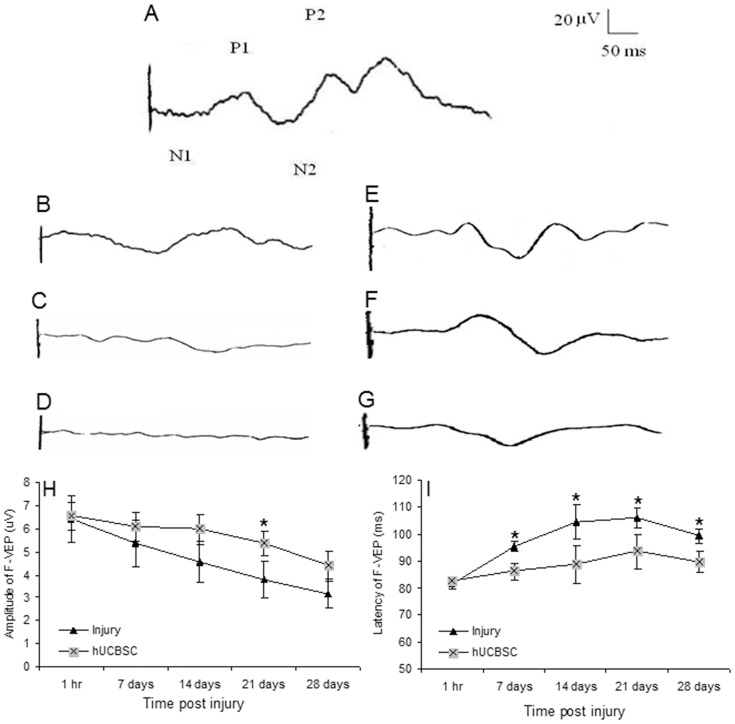
Measurement of optic nerve function by F-VEP. A) F-VEP in normal eye. B) Representative F-VEP in eye injured for 1 hr, 21 days (C), and 28 days (D). E) Representative F-VEP in the hUCBSC-transplanted eyes for 1 hr, 21 days (F), and 28 days (G). H) The amplitude of F-VEP at different time points in injury and hUCBSC transplantation groups. I) Latency of F-VEP at different time points in injury and hUCBSC transplantation groups. N = 5.

F-VEP graphics showed that in the sham-surgery group, F-VEP waveform was stable (Figure 1A) and no significant change was observed with time. The amplitude of F-VEP fluctuated between 12.1±1.5 and 13.5±1.6 μV, while the peak latencies fluctuated between 72.9±6.7 and 74.2±6.1 ms. In optic nerve injured rats, amplitude of F-VEP was lower and the waveform was wider. F-VEP latency became longer and amplitude became lower within the observation time (Figure 1B, C, D). At the same time point, F-VEP waveform was significantly higher in amplitude and shorter in latency in transplantation rats than in injured rats (Fig. 1E, F, G).

We further compared the P1 amplitude of F-VEP between injury group and transplantation group (Fig. 1H). Although the amplitude of F-VEP decreased with time in the transplantation group, the rate of decrease was lower than that of the injury group. At day 14, the amplitude of F-VEP was significantly higher in transplantation group than in the injury group. Significant statistical differences in the amplitude of F-VEP of optic nerve were observed between injury group and transplantation group at each time point (p<0.05 at 7 days; p<0.01 at 14, 21, and 28 days).

We also compared the peak latencies of F-VEP waveform between injury and transplantation group (Fig. 1I). The peak latencies increased over time in both the injury and transplantation groups. Starting from da 7, the peak latency in the transplantation group was shorter than in the injury group. Significant differences were observed beginning at day 7 post injury (p<0.05 for day 7; p<0.01 for day 14, 21, and 28).

### hUCBSC transplantation increased retinal ganglion cell number in optic nerve injured rats

**Figure 2:**
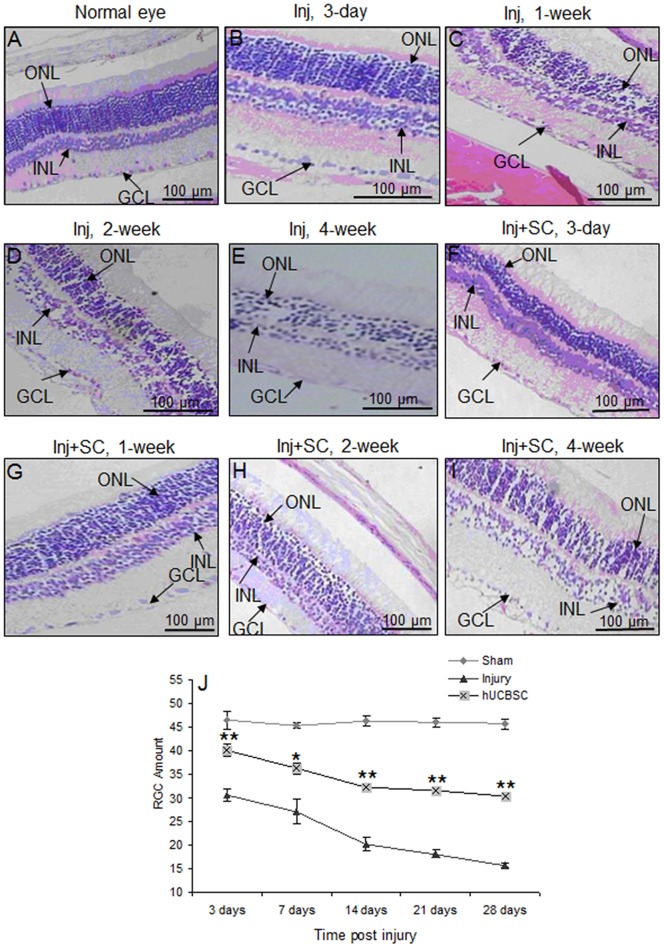
Detection of retinal ganglion cells. A) Normal eye staining. ONL: outer nuclear layer; INL: inner nuclear layer; GCL: ganglion cell layer. B–E) Staining for injured (Inj) eyes at 3-day, 1-week, 2-week, and 4-week post injury, respectively. F–I) Staining for hUCBSC transplanted (Inj+SC) eyes at 3-day, 1-week, 2-week and 4-week post surgery, respectively. J) Comparison of RGC number. ^*^ *p*<0.05, ^**^ *p*<0.001 *vs*. injury group. N = 5. A scale bar measures 100 microns.

To assess the neuroprotective role of hUCBSC transplantation, the morphological changes of RGCs were analyzed (Fig. 2A–I) and RGCs were counted (Fig. 2J). As shown in Fig. 2, RGC number was severely reduced after optic nerve was clamped for 30 seconds. Both the inner nuclear layer and outer nuclear layer become thinner, and obvious vacuolization and disorder in array were observed 28 days after injury. No difference in RGC number was observed between different time points in sham-surgery group. However, optic nerve injury significantly reduced RGC count at all of the observed time points post-surgery compared to the sham-surgery group. Transplantation of hUCBSCs significantly lowered the reduction in RGC count. Significant statistical differences were observed between sham-surgery, injury, and transplantation groups (*ps* <0.01).

### hUCBSC transplantation protected cells from apoptosis

**Figure 3:**
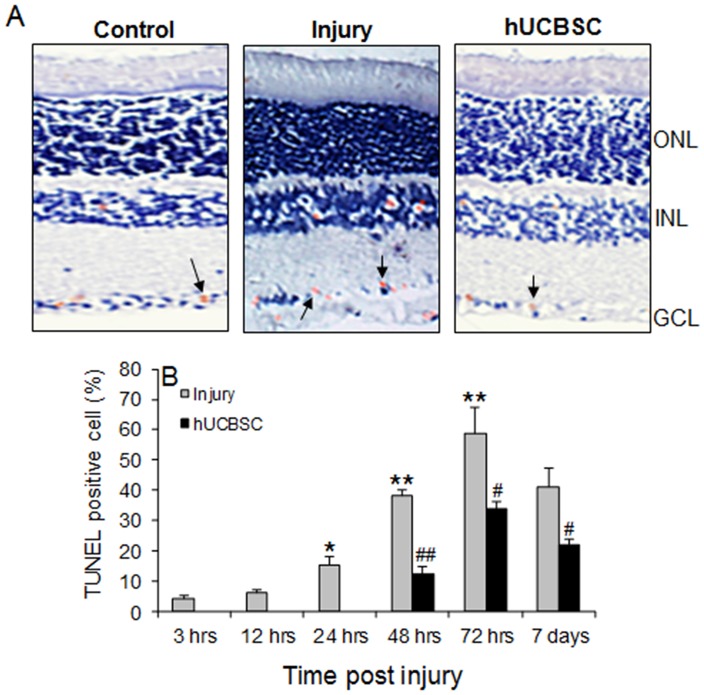
Detection of TUNEL-positive cells. A) Representative TUNEL staining of normal retina, injured retina 7 days after injury, and hUCBSC-transplanted retinas 7 days after surgery. TUNEL-positive cells were seen in inner nuclear layer and ganglion cell layer. Arrows indicated the TUNEL-positive cells (Brown). ONL: outer nuclear layer; INL: inner nuclear layer; GCL: ganglion cell layer. B) TUNEL-positive cells counting in retina in RGC layer at different time points in injury and hUCBSC transplanted rats. N = 5. ^*^ *p*<0.05, ^**^ *p*<0.01 *vs*. 3 hrs. ^#^ *p*<0.05, ^##^ *p*<0.01 *vs*. injury group.

Based on our finding on RGC survival, we further conducted TUNEL staining in injury and transplantation groups. In optic nerve injury group, few apoptosis-positive cells were seen in inner nuclear layer 3 hrs after optic nerve injury, but apoptosis-positive cells were obviously increased at 24 hrs. However, apoptosis appears to begin later in the ganglion cell layer than in the inner nuclear layer. Few apoptosis-positive cells were seen in ganglion cell layer 24 hrs after injury and the peak number was observed at 72 hrs. Also, clearly few apoptotic cells were seen in the core layer. Apoptotic cells obviously decreased at 7 days post-injury. In contrast, in hUCBSC transplantation rats, no obvious apoptotic cells were observed within 24 hrs. Apoptotic cells increased at 48 and 72 hrs post transplantation and decreased at 7 days after transplantation. Comparison of the total number of apoptosis-positive cells in retina between two groups (Fig. 3) showed significant differences at each time point (*ps* <0.01). This suggested that transplantation of hUCBSCs significantly protected retina cells from apoptosis induced by optic nerve injury.

### hUCBSC transplantation regulated GRP78 and CHOP expression

**Figure 4:**
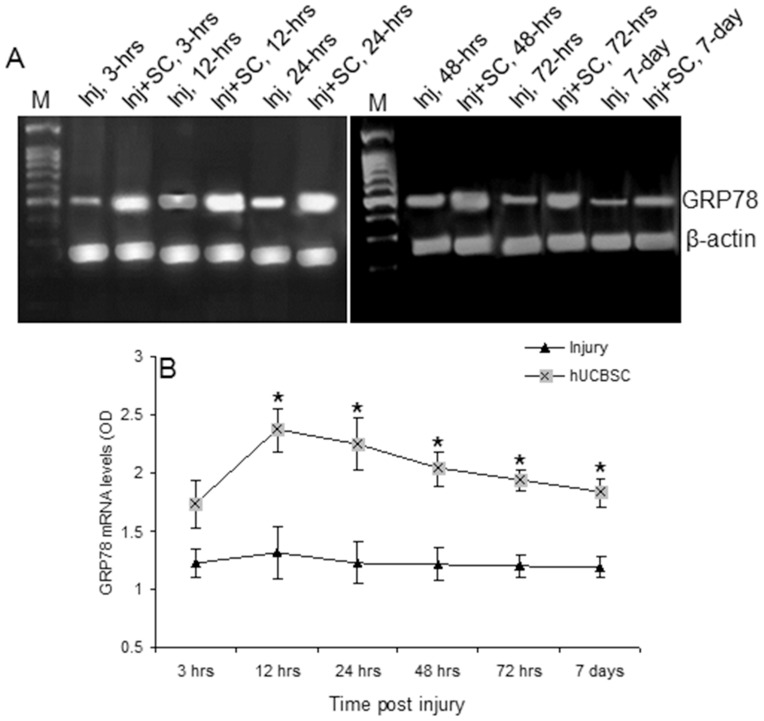
Detection of GRP78 mRNA expression. A) Representative RT-PCR of GRP78 expression. GRP78 mRNA expression was detected 3 hrs, 12 hrs, 24 hrs, 48 hrs, 72 hrs and 7 days post surgery in the injury (Inj) and hUCBSC transplantation (Inj+SC) groups. B) Comparison of the band-pixel values of GRP78 mRNA in injury and hUCBSC transplantation rats. ^*^ *p*<0.001 *vs*. injury group. N = 5.

**Figure 5:**
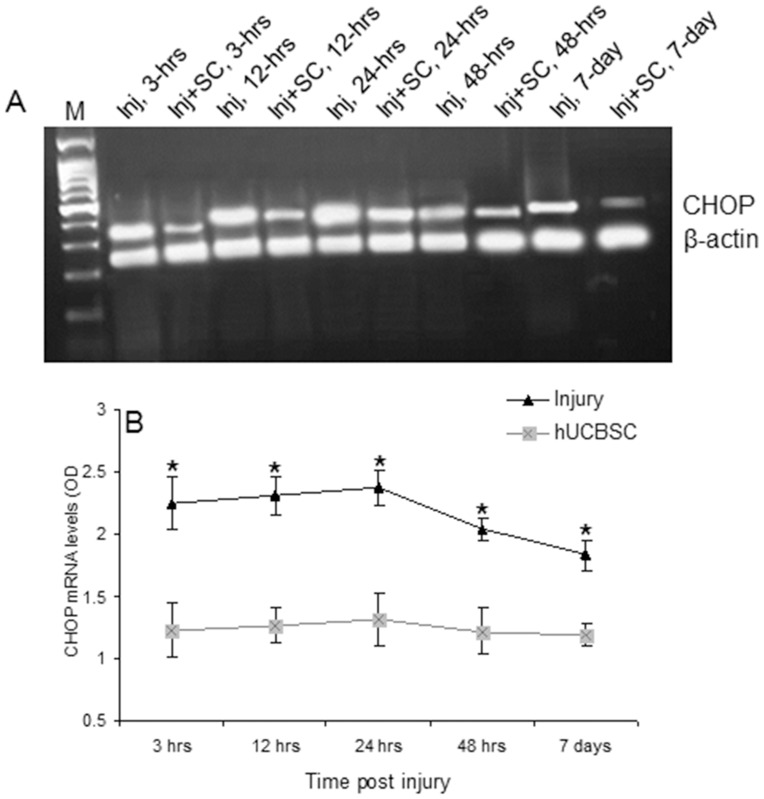
Detection of CHOP mRNA expression. A) Representative RT-PCR of CHOP expression. CHOP mRNA expression was detected 3 hrs, 12 hrs, 24 hrs, 48 hrs and 7 days post surgery in the injury (Inj) and hUCBSC transplantation (Inj+SC) groups. B) Comparison of the band-pixel values of CHOP mRNA in injury and hUCBSC transplantation rats. ^*^ *p*<0.001 *vs.* hUCBSC transplantation. N = 5.

Based on our findings on RGC survival and TUNEL staining, we further examined the dynamic change of GRP78 mRNA expression in injured rats and hUCBSC transplanted rats 3, 12, 24, 48, 72 hrs and 7 days post injury using RT-PCR. As shown in Fig. 4A, a relatively weak band corresponding to GRP78 was seen in retina tissue of injured rats 3 hrs after surgery with a peak at 12 hrs. In contrast, in transplantation rats, GRP78 mRNA expressions were obviously upregulated at each time point compared to the injury group. Comparison of the band-pixel value showed that GRP78 mRNA levels in retina of rats in transplantation group (Fig. 4B) showed significant increases at each time point compared to injury group (*ps* <0.01). Dynamic changes of CHOP mRNA expression in injured rats and hUCBSC transplanted rats were investigated at 3, 12, 24, 48 hrs and 7 days post injury using RT-PCR. As shown in Fig. 5A, a bright band corresponding to CHOP can be seen in the retina in the injury group and a relatively weak band was revealed in the hUCBSC transplantation group. Comparison of the band-pixel value showed that CHOP mRNA levels in retina transplanted with hUCBSC (Fig. 5B) showed significant decrease over time at each time point compared to the retina in injury group (*ps* <0.01). These results suggested that transplantation of stem cells significantly upregulated GRP78 and downregulated CHOP mRNA levels.

## Discussion

Currently, there is no standard therapeutic strategy available for the treatment of traumatic optic neuropathy (TON) in the clinic. Loss of RGC is a key cause for severe visual loss. Protection of RGC from death or replacing lost RGC may be a promising strategy for effective treatment of TON. Human umbilical cord blood stem cells (hUCBSC) have been proposed a potential treatment for conditions ranging from ischemic injury to neurodegenerative disease [4]. However, using hUCBSC transplantation to treat TON has not yet been established in the preclinical animal models. Particularly, how transplanted hUCBSCs participate in the optic nerve repairment is unclear. In this study we microinjected hUCBSC into the vitreous cavity of rats experiencing optic nerve injury. Transplantation of hUCBSCs significantly blunted a reduction in optic nerve function, increased RGC count, and decreased retinal cell apoptosis. The neuroprotective role of hUCBSC transplantation correlated with the upregulation of GRP78 expression and downregulation of CHOP expression in retinal cells. Our study suggested that transplantation of hUCBSC may be effective in treating TON.

Numerous reports have revealed that stem cells, including neuronal precursor cells and bone marrow stem cells, can play a protective role in a variety of central nervous system injury models [14]. Human umbilical cord blood is rich in hematopoietic stem cells and mesenchymal stem cells [14]. Mesenchymal stem cells have the ability to self-renew, proliferate, and differentiate into various cells or tissues when induced by certain factors. A recent study demonstrated that hUCBSCs can differentiate into retinal nerve cells at two weeks after the hUCBSC transplantation into mouse retina [15]. Therefore, hUCBSCs can participate in the optic nerve repair process by replacing lost RGC. In this study, optic nerve injury caused significant decrease in RGC count in both the inner and outer nuclear layers, as well as the RGC layer from day 3 after injury to the end of the observational period. Intravitreal injection of hUCBSCs significantly lowered the reduction in RGC count in the transplant group compared to the injury group (Fig. 2). However, this study provided no evidence whether the increased RGC count relates to neuroprotection of endogenous cells or the transplanted hUCBSCs differentiated into RGC. However, in the transplant group, significant more RGC count was observed on as early as day 3 and no difference was observed from day 14 to day 28. These observations do not fully support the hypothesis that the increased RGC in rats that received transplants was a result of hUCBSCs differentiation. Conversely, it implies that other protective mechanisms may also be involved in the lowering the reduction in RGC cell count.

Interestingly, Zwart et al transplanted mesenchymal cells isolated from hUCBSC into the vitreous cavity of SD rats, but they found that the mesenchymal cells were not able to differentiate into any type of nerve cells and also cannot bind to the retina [16]. However, transplantation of mesenchymal cells was neuroprotective and promoted regeneration of retinal nerve [16]. In contrast, studies demonstrated that mesenchymal cells can achieve the neuroprotective effect via secretion of several immunomodulatory and neurotrophic factors, including TGFβ1, CNTF/NT-3, and BDNF [17]. By considering that hUCBSCs contain both the hematopoietic stem cells and mesenchymal stem cells, our observation suggest that immunomodulatory and neurotrophic factors may also play a neuroprotective role. In this study, the peak TUNEL-positive cells were observed at 72 hrs and a reduction appeared at 1 week post injury in the injury group. In contrast, the RGC amount progressively decreased with time. RGC may die after traumatic optic nerve damage through apoptosis, necrosis and degeneration. The lack of correlation between RGC amount and TUNEL-positive cells suggested that other causes, such as degeneration might also be responsible for the RGC loss.

The most novel finding in this study is that transplantation of hUCBSCs interfered with ER stress-related proteins expression. Previous reports revealed that induction of ER stress-related proteins CHOP and/or GRP78 expression were involved in the tunicamycin or NMDA (a glutamate-receptor agonist) induced apoptosis in RGC *in vitro* and *in vivo* [18]. Tunicamycin is an inhibitor of *N*-linked glycosylation. Reduction of the *N*-glycosylation of proteins causes an accumulation of unfolded proteins in the ER and thus induces ER stress [11]. The accumulation of unfolded protein can stimulate GRP78 expression. GRP78 in turn works to restore folding in misfolded or incompletely assembled proteins [19]. In addition, NMDA induces CHOP protein and apoptosis in the ganglion cell layer and the inner plexiform layer [12], [20], suggesting that ER stress may be a factor in retinal injury. In this study, both GRP78 and CHOP were stably expressed from 3 hrs to 1 week after optic nerve injury. However, transplantation of hUCBSC significantly increased GRP78 expression and reduced CHOP expression at every time point. The increased GRP78 expression could restore folding in misfolded or incompletely assembled protein, therefore reduce apoptosis. CHOP is a central mediator of ER stress-induced apoptosis, and its expression under ER stress was reported to be up-regulated in proportion to the level of apoptotic cell death [20]. However, our study revealed that CHOP expression does not progressively increase with time after retinal nerve injury. In contrast, transplantation of hUCBSCs significantly downregulated CHOP expression at every time point. It may be possible that decreased expression in the proapoptotic protein CHOP directly reduced apoptosis. Therefore, our study implicated that transplantation of hUCBSCs may regulate ER stress, and subsequently decrease apoptosis in retinal neurons.

Repeatability, controllability, and detectability are critical characteristics of a novel nerve injury model. In this study, optic nerve injury was induced by directly clamping the optic nerve, which is easy to operate and can be accurately repeated. Importantly, the injury is controllable. Measurement of flash visual evoked potentials (F-VEP) is an objective and effective way to evaluate optic nerve function. In this study, transplantation of hUCBSCs significantly recovered optic nerve function as reflected by the change in F-VEP waveform. This provided solid evidence that the transplanted hUCBSCs protected neurons in the animal model and transplantation of hUCBSCs is a promising strategy for TON therapy. However, questions also rise from the current study. For example, how hUCBSCs were able to regulate GRP78 and CHOP expression remains unclear. Which plays the bigger role in RGC survival in the transplantation group: the differentiation of hUCBSCs into RGCs or the neuroprotective effect of hUCBSCs? Also, whether a combination of hUCBSC transplantation with classic therapy strategy, such as steroids application and surgical decompression, is more effective needs more study.

In conclusion, intravitreal transplantation of hUCBSCs is effective in recovering retinal nerve function through increasing RGC count. After transplantation, the increased RGC count, which could be the direct result of hUCBSC differentiation, is associated with a decrease in retinal cell apoptosis. Transplantation inhibits RGC apoptosis through inhibiting ER stress by upregulating GRP78 and downregulating CHOP expression. Our results strongly suggested that intravitreal transplantation of hUCBSCs is a promising strategy to treat traumatic optic neuropathy. However, the mechanism of the protective effect needs further in-depth study.
